# Associations between social vulnerability and functioning in older age, and the moderating role of optimism and self-efficacy

**DOI:** 10.1007/s40520-025-03077-6

**Published:** 2025-05-28

**Authors:** Anna-Maria Lahti, Tuija Mikkola, Anna Tirkkonen, Johan G. Eriksson, Mikaela von Bonsdorff

**Affiliations:** 1 Gerontology Research Center, Faculty of Sport and Health Sciences, University of Jyväskylä, Jyväskylä, PO box 35, 40014 Finland; 2 Folkhälsan Research Center, Topeliuksenkatu 20, Helsinki, 00250 Finland; 3 Clinicum, Faculty of Medicine, University of Helsinki, Helsinki, PO box 63, 00014 Finland; 4 Department of General Practice and Primary Health Care, University of Helsinki, Helsinki, PO Box 20, 00014 Finland; 5 Department of Obstetrics and Gynecology and Human Translational Research Programme, Yong Loo Lin School of Medicine, National University of Singapore, 1E Kent Ridge Road NUHS Tower Block, Level 12, Singapore, 119228 Singapore

**Keywords:** Social vulnerability, Physical functioning, Emotional functioning, Optimism, Self-efficacy

## Abstract

**Background:**

Although links between social factors, psychological characteristics and functioning have been established, interactions between social vulnerability and psychological characteristics impacting later-life functioning remain unclear. We investigated whether social vulnerability is associated with physical and emotional functioning and with the change in functioning over 5 years. Further, we studied whether optimism and self-efficacy moderate these associations.

**Methods:**

Physical and emotional functioning were measured in 2015 and 2020 using the SF-36 Health Survey in participants from the Helsinki Birth Cohort Study data (*n* = 1153, mean age 74y). Social vulnerability comprised several self-reported and register-based social indicators. We used linear mixed models to analyse the associations between social vulnerability and physical and emotional functioning, the change in functioning, and the moderating effects of optimism and self-efficacy.

**Results:**

Social vulnerability was inversely associated with the level of physical (β =-2.71, *p* < 0.001) and emotional functioning (β =-2.55, *p* < 0.001), as well as with the changes in physical functioning over the 5-year follow-up (β =-1.09, *p* = 0.003), but not with the decline in emotional functioning. Optimism and self-efficacy served as moderators by buffering the negative association of social vulnerability on emotional functioning, but not physical functioning. Optimism or self-efficacy did not moderate the change in physical or emotional functioning.

**Conclusion:**

By impacting social vulnerability we may be able to promote functioning in older age. Social and psychological characteristics need to be acknowledged when planning effective health interventions and services for older adults.

**Supplementary Information:**

The online version contains supplementary material available at 10.1007/s40520-025-03077-6.

## Introduction

Physical and emotional functioning are essential for maintaining autonomy, social participation, and independent living in older age. Physical functioning refers to an individual’s physical ability to perform day-to-day activities [1], and it has been shown to decline with age [2–4]. Emotional functioning is a complex concept, including the ability to experience, express, and manage emotion [5], often involving symptoms of emotional problems, and it appears to be fairly stable in older age [3, 6, 7]. Research has consistently shown that declines in physical and emotional functioning are strongly linked with adverse health outcomes, such as premature mortality [8, 9] and increased risk of long-term care [10, 11].

According to the Cumulative Disadvantage/Advantage (CDA) theory [12], social structures and social determinants accumulate throughout the life course, creating inequalities that may affect health. Social vulnerability refers to a situation where an individual’s social conditions form a disadvantage, making them more at risk of health problems [13]. The more adverse social circumstances a person has, the more vulnerable they are to adverse events, such as increased risk of cognitive decline, disability, need for long-term care, and mortality [13–17]. Social vulnerability is often measured through indices that combine various social factors, such as socioeconomic status (SES), education, social participation and loneliness [15, 18, 19]. Older age increases the risk of social vulnerability [16, 20] as older people are more likely to be widowed, lonely and/or living alone. Hence, societies with increasing life expectancy will likely see a rise in socially vulnerable populations. Understanding the associations between social vulnerability and functioning can help identify people at risk, enabling early intervention and prevention of declines in functioning. This highlights the need for research that investigates how social vulnerability impacts physical and mental functioning in later life, particularly as social vulnerabilities may compound over time, as suggested by the CDA theory.

In addition to social factors, psychological characteristics like optimism and self-efficacy are known to influence health outcomes. *Optimism* is defined as a general expectation that positive things will happen even when things are hard [21]. Previous research has demonstrated the protective role of optimism in aging populations, as higher optimism is linked to better health outcomes, such as longevity, healthy aging [22–26], better cognitive functioning [27], less experienced pain [28] and better physical and emotional functioning [22, 29, 30] among older adults. In addition to health, optimism is also associated with social factors like higher SES and social support [31]. Optimistic people tend to have better social resources, such as more social support and social networks [21]. *Self-efficacy* refers to an individual’s belief in their capacity to achieve goals. People with high self-efficacy experience difficult tasks as challenges that they aim to master, while people with low self-efficacy may experience them as threats that need to be avoided [32]. Self-efficacy develops most effectively through experiences of success, but also by receiving social encouragement, where others verbally affirm one’s capability to succeed [32]. Self-efficacy is associated with SES [33] and has been shown to predict lower levels of loneliness, pain and better mental health [34–36].

The protective nature of optimism and self-efficacy suggests that they may act as moderating factors in the relationship between social vulnerability and functioning, serving as indicators of resilience [37] that shield against the negative impact of social vulnerability. The Theory of Social Stress [38] establishes that social conditions generate stress that unequally affects populations based on their social circumstances. Optimism and self-efficacy may provide a buffering effect against stress and stressful experiences caused by social vulnerability. Previous research demonstrates that both optimism and self-efficacy are associated with lower stress levels [39–42]. When these psychological resources are present, individuals may be able to withstand higher levels of stress without experiencing significant declines in functioning. Conversely, when these characteristics are weak, the negative effects of social vulnerability may be stronger, contributing to poorer functioning outcomes.

In this study, we investigated whether social vulnerability is associated with physical and emotional functioning in older age and with the decline in functioning during a 5-year follow-up. In addition, we tested whether optimism and self-efficacy moderate the relationship between social vulnerability and functioning. We hypothesize that social vulnerability has a negative association with the level and change of physical and emotional functioning. We expect optimism and self-efficacy to serve as moderators between social vulnerability and functioning by buffering the negative effect of social vulnerability on both the levels of physical and emotional functioning and changes over time.

## Methods

### Study population

This study used data from the Helsinki Birth Cohort Study, a longitudinal study of 13,345 individuals born between 1934 and 1944 at the Helsinki University Central Hospital (HUCH) or Helsinki City Maternity Hospital in Helsinki, Finland. In 2000, a random sample of 2902 individuals born at HUCH were invited to participate in a clinical examination between the years 2001 and 2004, of which 2003 individuals (69%) attended (mean age = 61.5y). This is considered the original sample of the study. In 2015, a postal questionnaire was sent out to the participants from the clinical baseline visit who were alive and traceable (*n* = 1577). Of them, 1153 returned the questionnaire, representing 58% of the original sample. In 2020, another follow-up survey was sent out to participants who were alive and traceable of the clinical baseline cohort (*n* = 1361), of which 926 participated, representing 46% of the original sample. All participants provided written informed consent.

### Study variables

*Physical and emotional functioning* were assessed in 2015 and 2020 using the validated and reliable Finnish version of the Short Form Health Survey (SF-36) [43, 44]. The SF-36 includes 36 items measuring health and functioning across eight domains. All the domains have reported good reliability (Cronbach’s alpha at least 0.8) [43] im previous research. We used the ‘physical function’ and ‘emotional wellbeing’ domains as the primary outcomes, which both showed good reliability (Cronbach’s alphas of 0.9 and over 0.8, respectively) in this study. The ‘physical function’ domain comprises 10 items assessing whether the respondent’s health limits his/her ability to pursue daily activities such as walking 2 km, climbing stairs or lifting or carrying groceries. Answer options were (1) Yes, limits a lot, (2) Yes, limits a little and (3) No, does not limit at all. The ‘emotional wellbeing’ domain comprises 5 items which captures depressive and anxiety symptoms with questions such as: “How much of the time have you been a happy person”, or “How much of the time have you felt very nervous”. Answer options were on a 6-point Likert scale ranging from “All of the time” to “None of the time”. The domains were standardized using the means and standard deviations of the US reference population and weighted with factor score coefficients from the same reference population [45].

*Social vulnerability* was measured using a cumulative deficit approach [13, 18]. We used 14 social indicators to calculate an index: educational attainment, highest occupational status during adulthood (i.e. social class), income, social contacts with family, friends and acquaintances, participation in leisure activities (hobbies and culture) and voluntary activities (voluntary work and work at church or associations etc.), marital status, loneliness, home ownership and having children. Correlations of the social vulnerability index and the social variables are reported in Supplementary Table 1. Educational attainment was self-reported in 2001–2004. Occupational status and having children were register-based information from the year 2000 and were derived from Statistics Finland. All other variables were self-reported from the postal survey in 2015. The items were chosen based on prior studies indicating their relevance, and the aim was to capture a range of factors that reflect a person’s social circumstances [13, 18, 46]. All social variables were coded between 0 and 1. Variables with a Likert scale answer options were scaled in the interval 0–1 (Supplementary Table 2). Scaled variables were then summed to an index variable with a possible range from 0 to 14. Higher scores indicate higher social vulnerability.

*Optimism* was measured using the Life Orientation Test Revised (LOT-R), a validated psychological instrument for assessing optimism [47, 48]. The reliability of the measure in this study was sufficient with a Cronbach’s alpha of 0.70. The test consisted of 10 statements with a 5-point Likert-scale answer options, e.g. “In uncertain times, I usually expect the best” and “If something can go wrong for me, it will”. For an overall score, the items were summed into a sum variable with a possible range from 0 to 24. A higher score indicated higher optimism.

*Self-efficacy* was assessed using the General Self-Efficacy Scale (GSE) [49], which is widely used and validated and has an internal reliability (Cronbach’s alpha) of 0.88 in this study. GSE comprised 10 statements concerning the respondent’s ability to act in various situations and how they trust their own abilities, such as “If I am in trouble, I can usually think of a solution” and “I can usually handle whatever comes my way”. Answer options were on a 4-point Likert scale. The total score was calculated by summing all items, and the total score ranged between 10 and 40, with a higher score indicating higher self-efficacy. A mean score was calculated by dividing the total score with the number of available items.

Age and sex were included as covariates to control for their effect on functioning. Chronic diseases were treated as integral components of functioning rather than confounding variables in our main analysis. However, chronic diseases that developed before social vulnerability could potentially confound the tested relationships. Therefore, we conducted an additional sensitivity analysis (Supplementary Table 4) where chronic diseases was included as a covariate. Chronic diseases were self-reported in the survey in 2015 and included 15 diseases or symptoms that were combined to a sum variable: hypertension, heart failure, arrhythmia, angina pectoris, myocardial infarction, claudication, osteoporosis, stroke, cancer, depression, asthma, emphysema, diabetes, dementia and rheumatic disorders. As adjusting for chronic diseases did not change the results in the main analysis, we continued to run the moderation analyses adjusting for only age and sex.

### Statistical methods

Baseline characteristics of the study population were analysed using SPSS IBM version 28.0 (SPSS, Armonk, NY, IBM Corp). Before further analyses, we assessed the correlations among the main variables using the Pearson correlation test (Supplementary Table 3). We ran an attrition analysis comparing the clinical baseline data of participants who returned the surveys, who did not return the surveys, who died, and who were not traceable, using ANOVA for continuous variables and chi-square test for categorical variables (Supplementary Table 5). We then ran linear mixed models, which account for invidual level variation, using R version 4.4.1 with package lme4 to investigate the longitudinal associations between social vulnerability and physical and emotional functioning, and the moderation effects of optimism and self-efficacy. The analyses were adjusted for age and sex.

## Results

### Descriptive results

Characteristics of the study population are shown in Table 1. The mean age of the participants was 74 years, and 44% of them were men, most (69%) were married or cohabiting, had children (89%) and owned their home (86%). Almost half (46%) of the participants had low official as their occupational status. Education was more evenly spread out: 24% had a university degree, 29% an elementary level education, 19% had vocational education and 29% had attended basic education like middle school. Most of the participants were in contact with their family and friends weekly (61% and 54%, respectively), while 70% of them were in contact with acquaintances only monthly or less. A quarter (26%) of the participants reported feelings of loneliness sometimes or often. The participants attended hobbies most often weekly (37%) or less (41%), and attended other activities such as theaters and concerts mostly monthly (45%) or less (40%). Most of the participants did not do voluntary work or act in a position of trust (72% and 75%, respectively). Their mean social vulnerability index was 5,8 (SD 2,7). Mean score for physical functioning in 2015 was 76,6 (SD 22,7) and 74,3 (SD 24,0) five years later in 2020. Mean score for emotional functioning in 2015 was 81,3 (SD 15,9) and 78,8 (SD 16,3) in 2020. Mean scores for optimism and self-efficacy were 17,4 (SD 3,9) and 3,0 (SD 0,4), respectively.

**Table 1:** Participants characteristics

	Mean	(SD)
**Age** *(n = 1153)*	74,0	(2,7)
**Social vulnerability index** *(n = 1153)*	5,8	(1,7)
**Physical functioning in 2015** *(n = 1151)*	76,6	(22,7)
**Physical functioning in 2020** *(n = 806)*	74,3	(24,0)
**Emotional functioning 2015** *(n = 1148)*	81,3	(15,9)
**Emotional functioning 2020** *(n = 806)*	78,8	(16,3)
**Optimism** *(n = 1152)*	17,4	(3,9)
**Self-efficacy** *(n = 1145)*	3,0	(0,4)

	*n*	*(%)*
**Sex** *(n = 1153)*		
Men	503	(44)
**Education** *(n = 1150)*		
University degree	272	(24)
Elementary school	335	(29)
Vocational school	214	(19)
Folk school, middle school etc.	329	(29)
**Occupational status** *(n = 1153)*		
High official	195	(17)
Low official	525	(46)
Self-employed	105	(9)
Labourers	328	(28)
**Household income per month** *(n = 1135)*		
4000 € or more	215	(19)
2000–3999 €	618	(55)
Under 2000 €	302	(27)
**Married or cohabiting** *(n = 1146)*	791	(69)
**Has children** *(n = 1153)*	1020	(89)
**Home ownership** *(n = 1140)*	984	(86)
**Loneliness** *(n = 1146)*		
Never or rarely	842	(73)
Sometimes or often	304	(26)
**Contact with family** *(n = 1149)*		
Everyday	224	(20)
Weekly	704	(61)
Monthly or less	221	(19)
**Contact with friends** *(n = 1149)*		
Everyday	109	(10)
Weekly	616	(54)
Monthly or less	424	(37)
**Contact with acquaintances** *(n = 1148)*		
Everyday	38	(3)
Weekly	309	(27)
Monthly or less	801	(70)
**Acting in a position of trust (e.g. church**,** councils)***(n = 1140)*		
Daily or weekly	79	(7)
Monthly or less	209	(18)
Not at all	852	(75)
**Voluntary work in an organization** *(n = 1143)*		
Daily or weekly	86	(8)
Monthly or less	230	(20)
Not at all	827	(72)
**Hobbies**,** clubs etc.***(n = 1149)*		
Daily	138	(12)
Weekly	419	(37)
Monthly	120	(10)
Less	472	(41)
**Theatres**,** cafes**,** concerts etc.***(n = 1144)*		
Daily	44	(4)
Weekly	130	(11)
Monthly	518	(45)
Less	452	(40)

The attrition analyses revealed that the participants who did not return the survey in 2015 or in 2020 were slightly older and had lower physical and emotional functioning at the clinical baseline in 2001–2004 (Supplementary Table 5). In addition, they had lower occupational status and they were less optimistic and had lower self-efficacy.

### Social vulnerability and functioning

In the linear mixed models analysis (adjusted for age and sex) emotional functioning showed a significant decline from 2015 to 2020 (β =-5.86 *p* < 0.001), while physical functioning did not statistically significantly change (Table 2). Social vulnerability was associated with both physical and emotional functioning. Higher social vulnerability was associated with a lower level of physical functioning (β =-2.71,*p* < 0.001) and had a negative impact on the changes in physical functioning over the 5-year follow-up (β =-1.09, *p* = 0.003). Higher social vulnerability was significantly associated with lower level of emotional functioning (β =-2.55, *p* < 0.001), but not with the decline in emotional functioning over the 5-year period (Table 2).

**Table 2:** Coefficients for the associations of social vulnerability index and physical and emotional functioning using linear mixed models. Adjusted for age and sex

		β	SE	*p*-value
Physical functioning				
	Time (2015 to 2020)	1.02	2.11	0.627
	SVI	-2.71	-0.60	< 0.001
	Time*SVI	-1.09	0.36	0.003
Emotional functioning				
	Time (2015 to 2020)	-5.86	1.68	< 0.001
	SVI	-2.55	0.47	< 0.001
	Time*SVI	0.42	0.29	0.143

SE = Standard Error, SVI = social vulnerability index

### Moderation analyses

Optimism had a moderating effect on the association between social vulnerability and emotional functioning (β = 1.86, *p* < 0.001), but not physical functioning (Table 3). This indicates that higher optimism significantly decreased the strength of the inverse association between social vulnerability and emotional functioning. Optimism did not show a moderation effect on the association between social vulnerability and the change in physical or emotional functioning over the 5-year period.

**Table 3:** Coefficients for the associations of social vulnerability, optimism and physical and emotional functioning using linear mixed models. Adjusted for age and sex

		β	SE	*p*-value
Physical functioning				
	Time (2015 to 2020)	1.24	2.18	0.570
	SVI	-2.07	0.62	< 0.001
	Time*SVI	-1.12	0.37	0.003
	SVI*Optimism	0.96	0.59	0.103
	Time*SVI*Optimism	0.09	0.25	0.799
Emotional functioning				
	Time (2015 to 2020)	-4.93	1.72	0.004
	SVI	-1.07	0.46	0.020
	Time*SVI	0.24	0.29	0.422
	SVI*Optimism	1.86	0.43	< 0.001
	Time*SVI*Optimism	-0.33	0.28	0.247

SE = Standard Error, SVI = social vulnerability index

Similarily, self-efficacy showed a moderation effect on the association between social vulnerability and emotional functioning (β = 1.01, *p* = 0.03) but not physical functioning (Table 4), suggesting that higher self-efficacy reduced the strength of the inverse association between social vulnerability and emotional functioning. Self-efficacy did not moderate the association between social vulnerability and the change in physical or emotional functioning over 5 years.

**Table 4:** Coefficients for the associations of social vulnerability, self-efficacy and physical and emotional functioning using linear mixed models. Adjusted for age and sex

		β	SE	*p*-value
Physical functioning				
	Time (2015 to 2020)	0.006	2.15	0.998
	SVI	-2.73	0.62	< 0.001
	Time*SVI	-0.873	0.37	0.020
	SVI* Self-efficacy	-0.20	0.59	0.735
	Time*SVI* Self-efficacy	0.48	0.37	0.191
Emotional functioning				
	Time (2015 to 2020)	-5.83	1.72	< 0.001
	SVI	-1.85	0.47	< 0.001
	Time*SVI	0.42	0.29	0.150
	SVI* Self-efficacy	1.01	0.45	0.030
	Time*SVI* Self-efficacy	-0.07	0.29	0.817

SE = Standard Error, SVI = social vulnerability index

## Discussion

This study examined how social vulnerability is associated with physical and emotional functioning in older adults, and whether optimism and self-efficacy moderate these associations. The findings revealed that higher social vulnerability was associated with poorer physical and emotional functioning, and negatively affected changes in physical, but not emotional functioning over time. Both optimism and self-efficacy moderated the association between social vulnerability and emotional functioning, but not physical functioning. Neither optimism nor self-efficacy moderated the effect of social vulnerability on the change in physical or emotional functioning. To the best of our knowledge, no previous studies have focused on the association between social vulnerability and functioning in old age, and on the role of optimism and self-efficacy.

Our results on the association between social vulnerability and functioning are in accordance with previous studies, as higher level of social vulnerability has been inversely associated with several indicators of health in later life, such as disability, mortality and cognitive decline [13, 17, 18]. Social vulnerability had a negative influence on how physical functioning changed over time, as higher social vulnerability may predict a steeper decline in physical functioning. Due to their socially vulnerable position, these individuals may be more susceptible to chronic stress or face more barriers in accessing healthcare, which may then impact their health. When these social stressors and barriers accumulate over time, as suggested by the CDA theory [12, 50], they create a compounding effect, which may explain why social vulnerability is associated not only with lower physical functioning but also with the change in physical functioning. However, social vulnerability did not seem to affect the decline of emotional functioning, which implies that although social vulnerability is linked to lower overall emotional functioning, it may not accelerate the decline over time. Perhaps the decline of emotional functioning follows similar patterns regardless of social vulnerability, or socially vulnerable individuals may adapt to the chronic stressors and thus prevent the steeper decline of emotional functioning. It is possible that we were not able to capture underlying factors, such as resilience traits, that mitigate the impact of social vulnerability on the decline of emotional functioning over time.

Optimism and self-efficacy moderated the association between social vulnerability and emotional functioning. These psychological characteristics appear to serve as protective factors that buffer against the negative effects of social vulnerability. Individuals with higher levels of optimism and self-efficacy may be able to maintain their emotional functioning better despite experiencing social disadvantages or challenges. These individuals may have more effective coping strategies and be more resilient to chronic stressors [21, 37, 51–53]. They may face adverse situations in more positive ways, finding them as opportunities or challenges, when others might find them as threats [51, 52]. For example, when individuals have high levels of optimism or self-efficacy, they may view adverse situations as temporary rather than permanent barriers, show more effort and persistence in trying to eliminate or reduce the stressors they are facing, or be more motivated to maintain, for example, healthy behaviors, and this way potentially reduce the negative impact of social vulnerability [51, 53, 54].

Interestingly, our results showed that self-efficacy and optimism did not moderate the relationship between social vulnerability and physical functioning. This finding is slightly contradictory with previous research, as previous studies have linked self-efficacy and optimism with better physical health outcomes and healthier lifestyles [51, 55]. Our results suggest that while psychological characteristics may buffer the emotional impacts of social vulnerability, their protective effect may not extend to physical functioning. While self-efficacy and optimism are beneficial for overall functioning, psychological characteristiscs may not be powerful enough to prevent the cumulative disadvantages that accelerate functional decline. Psychological characteristics might help individuals cope with challenges, but they cannot overcome the fundamental limitations in resources, healthcare access barriers, or physiological processes associated with aging. Similar reasons may underlie the findings on optimism or self-efficacy not moderating the change in either physical or emotional functioning.

By impacting social factors, such as social networks and social participation, it may be possible to reduce social vulnerability and therefore reduce disparities in emotional and physical functioning in older age. However, social disadvantages do not directly determine functioning in older age, since protective psychological characteristics like optimism and self-efficacy play a significant role. By acknowledging and promoting optimism and self-efficacy among older adults, we can potentially alleviate the impact of social vulnerability on functioning in older age, which in turn may improve health and well-being in older adults. Social circumstances and psychological characteristics may define how personalized interventions aimed at maintaining functioning are best designed and implemented.

The strengths of our study include the use of longitudinal data from a well-defined cohort, including validated and reliable measures on physical and emotional functioning, optimism and self-efficacy. The use of these measures makes our results comparable with other studies using the same items. In addition, we were able to include follow-up data on functioning over 5 years, which enabled the investigation of short-term change in functioning.

This study has limitations. First, as often happens in studies focusing on the older population, those with worse health and functioning often drop out or do not participate in the first place. As the attrition analyses showed, our study sample was healthier and slightly younger than the participants who did not participate, died or were not reached. Second, our follow-up time was relatively short, and any long-term conclusions on the associations cannot be drawn from our study. Third, physical and emotional functioning were self-reported, and optimistic individuals might rate their functioning higher than those who are less optimistic. However, self-assessment reflects how individuals perceive their own health, which is an important factor in overall well-being and often correlates strongly with objective health outcomes [56].

Furthermore it must be noted, that both health selection and social causation are likely to create health inequalities [57]. Our results lean on the ‘social causation’ hypothesis, in which better social circumstances are more beneficial to health than worse social conditions [57]. However, it is possible that the relationship between social vulnerability and physical and emotional functioning is bidirectional, similarly to social vulnerability and frailty [58]. In this case, our results could be interpreted through the ‘health selection’ hypothesis, in which people with good health are able to achieve more favorable social circumstances than people with poor health [57]. Thus, poorer physical and emotional functioning would be associated with higher social vulnerability. The role of psychological characteristics needs to be further studied.

## Conclusions

With this study we were able to better understand how social vulnerability relates to emotional and physical functioning in old age, and how psychological characteristics may shape the relationship. Reducing social vulnerability may improve older people’s functioning, and protective psychological characteristics may help in buffering the negative effects of social vulnerability. Future studies should test the moderation over longer time periods as well as with objective measures of functioning.

## Electronic supplementary material

Below is the link to the electronic supplementary material.

Supplementary Material 1

## Data Availability

The datasets generated and/or analyzed during the current study are available from the corresponding author on a reasonable request.
