# Supplementary material for: Associations between social vulnerability and functioning in older age, and the moderating role of optimism and self-efficacy
Source: Aging Clin Exp Res. 2025 May 28;37(1):173. doi: 10.1007/s40520-025-03077-6 (PMC12116706; doi:10.1007/s40520-025-03077-6)
Supplement: Supplementary file 1 — Supplementary Material 1 [file 40520_2025_3077_MOESM1_ESM.docx]

**Supplementary table 1. Correlations of the social vulnerability index and the scaled social variables included in the index.**

|  | **Social vulnerability index** | |
| --- | --- | --- |
|  | Pearson Correlation | p-value |
| Income | 0.598 | <0.001 |
| Education | 0.522 | <0.001 |
| Marital status | 0.449 | <0.001 |
| Occupation | 0.448 | <0.001 |
| Social participation in group activities | 0.444 | <0.001 |
| Associations, organizations | 0.410 | <0.001 |
| Housing | 0.401 | <0.001 |
| Social participation in theatres etc. | 0.392 | <0.001 |
| Loneliness | 0.383 | <0.001 |
| Voluntary work | 0.379 | <0.001 |
| Contact with acquaintances | 0.262 | <0.001 |
| Contact with friends | 0.257 | <0.001 |
| Children | 0.233 | <0.001 |
| Contact with family | 0.207 | <0.001 |

**Supplementary table 2. Coding of the variables in the social vulnerability index**

| **Variable** | **Coding** | **Source** |
| --- | --- | --- |
| Education | 0=University degree, 0.33= Elementary school, 0.66=Vocational school and 1=Folk school, middle school etc. | Survey from clinical baseline in 2001-2003 |
| Occupation | 0=High official, 0.33=Low official, 0.66=Self-employed and 1=Laborers | Statistics Finland 2000 |
| Household income in euros per month | 0=Over 5000, 0.2=4000-5000, 0.4=3000-3999, 0.6=2000-2999, 0.8=1000-1999 and 1= less than 1000. | Postal survey in 2015 |
| Contact with family or children | 0=Every day, 0.2= Every week, 0.4=Every month, 0.6=Couple of times a year, 0.8=Seldom or not at all and 1=I don’t have family | Postal survey in 2015 |
| Contact with close friends | 0=Every day, 0.2= Every week, 0.4=Every month, 0.6=Couple of times a year, 0.8=Seldom or not at all and 1=I don’t have close friends | Postal survey in 2015 |
| Contact with acquaintances | 0=Every day, 0.2= Every week, 0.4=Every month, 0.6=Couple of times a year, 0.8=Seldom or not at all and 1=I don’t have acquaintances. | Postal survey in 2015 |
| Group activities or clubs (choirs, sports groups, day centers etc.) | 0=Every day, 0.166=Every week, 0.33=2-3 times a month, 0.5=Once a month, 0.76=Couple of times a year, 0.83= Seldom and 1=Not at all. | Postal survey in 2015 |
| Frequent concerts, theatres, movies, art exhibitions, coffee shops etc. | 0=Every day, 0.166=Every week, 0.33=2-3 times a month, 0.5=Once a month, 0.76=Couple of times a year, 0.83= Seldom and 1=Not at all. | Postal survey in 2015 |
| Volunteering through an organization or association | 0=Every day, 0.166=Every week, 0.33=2-3 times a month, 0.5=Once a month, 0.76=Couple of times a year, 0.83= Seldom and 1=Not at all. | Postal survey in 2015 |
| Attending or holding a position of trust or a management role or similar councils, church or associations | 0=Every day, 0.166=Every week, 0.33=2-3 times a month, 0.5=Once a month, 0.76=Couple of times a year, 0.83= Seldom and 1=Not at all. | Postal survey in 2015 |
| Marital status | 0=Married or cohabiting and 1=Not married. | Postal survey in 2015 |
| Loneliness | 0=Never or rarely, 0.5=Sometimes and 1=Often. | Postal survey in 2015 |
| House ownership | 0= Owner-occupied and 1=Not owning. | Postal survey in 2015 |
| Children | 0=Yes and 1=No. | Statistics Finland 2000 |

**Supplementary table 3. Pearson correlation coefficients between main variables (n=1140-1150)**

|  | 1. | 2. | 3. | 4. |
| --- | --- | --- | --- | --- |
| 1.Physical functioning |  |  |  |  |
| 2. Emotional functioning | 0.35^***^ |  |  |  |
| 3. Optimism | 0.23^***^ | 0.51^***^ |  |  |
| 4. Self-efficacy | 0.17^***^ | 0.42^***^ | 0.44^***^ |  |
| 5. Social vulnerability index (SVI) | -0.31^***^ | -0.24^***^ | -0.27^***^ | -0.20^***^ |

***p<0.001

**Supplementary table 4**. Coefficients for the associations of social vulnerability index and physical and emotional functioning using linear mixed

models. Adjusted for age, sex (Model 1) and additionally chronic diseases (Model 2).

|  |  | Model 1 |  | Model 2 |  |
| --- | --- | --- | --- | --- | --- |
|  |  | β (SE) | p-value | β (SE) | p-value |
| Physical functioning |  |  |  |  |  |
|  | Time (2015 to 2020) | 1.02 (2.11) | 0.627 | 1.10 (2.10) | 0.600 |
|  | SVI | -2.71 (-0.60) | <0.001 | -1.57 (0.59) | 0.008 |
|  | Time*SVI | -1.09 (0.36) | 0.003 | -1.13 (0.36) | 0.002 |
| Emotional functioning |  |  |  |  |  |
|  | Time (2015 to 2020) | -5.86 (1.68) | <0.001 | -5.76 (1.68) | <0.001 |
|  | SVI | -2.55 (0.47) | <0.001 | -2.19 (0.47) | <0.001 |
|  | Time*SVI | 0.42 (0.29) | 0.143 | 0.38 (0.29) | 0.187 |

SE=Standard Error, SVI= social vulnerability index

**Supplementary table 5**. Clinical baseline (2001-2004) characteristics of the study population according to status in 2015 and 2020.

|  |  | | **Status in 2015** | | | |  | | |  | |  |
| --- | --- | --- | --- | --- | --- | --- | --- | --- | --- | --- | --- | --- |
| **Clinical baseline characteristics** | Returned survey  (n=1153) | | Did not return  survey (n=424) | | Died (n=254) | | No contact, survey not sent (n=172) | | | p | |  |
| Age at baseline *mean (SD)* | 61,3 | (2,8) | 61,9 | (3,1) | 62,0 | (3,1) | | 61,6 | (3,0) | | <0.001 | |
| Physical functioning *mean (SD)* | 85,1 | (16,9) | 78,6 | (20,0) | 74,0 | (24,4) | | 82,8 | (17,9) | | <0.001 | |
| Emotional functioning *mean (SD)* | 81,6 | (14,8) | 79,4 | (16,0) | 80,7 | (16,5) | | 82,1 | (13,4) | | 0.050 | |
| Optimism *mean (SD)* | 17,8 | (3,7) | 17,0 | (3,9) | 17,1 | (4,2) | | 17,0 | (3,7) | | <0.001 | |
| Self-efficacy *mean (SD)* | 3,0 | (0,4) | 2,9 | (0,4) | 3,0 | (0,4) | | 2,9 | (0,4) | | 0.021 | |
| Men *n (%)* | 503 | (44) | 189 | (45) | 146 | (59) | | 83 | (48) | | <0.001 | |
| Married *n (%)* | 893 | (78) | 308 | (74) | 173 | (71) | | 129 | (77) | | 0.068 | |
| Social class *n (%)* |  |  |  |  |  |  | |  |  | | <0.001 | |
| High official | 195 | (17) | 41 | (10) | 21 | (9) | | 28 | (16) | |  | |
| Low official | 525 | (46) | 166 | (39) | 93 | (38) | | 69 | (40) | |  | |
| Self-employed | 105 | (9) | 41 | (10) | 31 | (13) | | 9 | (5) | |  | |
| Labourers | 328 | (28) | 173 | (41) | 100 | (41) | | 65 | (38) | |  | |
|  |  |  |  |  |  |  | |  |  | |  | |
|  |  |  | **Status in 2020** | | | | |  |  | |  | |
|  | Returned survey  (n=926) | | Did not return  survey (n=435) | | Died (n=473) | | No contact, survey not sent (n=169) | | | p | |  |
| Age at baseline *mean (SD*) | 61,1 | (2,7) | 61,8 | (2,9) | 62,2 | (3,2) | | 61,6 | (3,9) | | <0.001 | |
| Physical functioning *mean (SD)* | 86,5 | (15,2) | 80,4 | (18,2) | 75,1 | (24,1) | | 81,9 | (19,1) | | <0.001 | |
| Emotional functioning *mean (SD*) | 81,9 | (14,5) | 79,3 | (15,9) | 81,2 | (15,8) | | 80,7 | (15,2) | | 0.035 | |
| Optimism me*an (SD)* | 17,9 | (3,7) | 16,8 | (3,8) | 17,3 | (4,0) | | 17,0 | (4,0) | | <0.001 | |
| Self-efficacy *mean (SD)* | 3,0 | (0,4) | 2,9 | (0,4) | 3,0 | (0,4) | | 2,9 | (0,4) | | <0.001 | |
| Men *n (%)* | 396 | (42,8) | 179 | (41) | 274 | (59) | | 72 | (43) | | <0.001 | |
| Married  *n (%)* | 723 | (79) | 323 | (76) | 331 | (72) | | 126 | (76) | | 0.031 | |
| Social class  *n (%)* |  |  |  |  |  |  | |  |  | | <0.001 | |
| High official | 167 | (18) | 45 | (10) | 49 | (11) | | 24 | (14) | |  | |
| Low official | 423 | (46) | 179 | (41) | 167 | (36) | | 84 | (50) | |  | |
| Self-employed | 100 | (11) | 27 | (6) | 47 | (10) | | 12 | (7) | |  | |
| Labourers | 235 | (25) | 183 | (42) | 199 | (43) | | 49 | (29) | |  | |
